# Microcystin Content in Phytoplankton and in Small Fish from Eutrophic Nyanza Gulf, Lake Victoria, Kenya

**DOI:** 10.3390/toxins10070275

**Published:** 2018-07-03

**Authors:** Benard Mucholwa Simiyu, Steve Omondi Oduor, Thomas Rohrlack, Lewis Sitoki, Rainer Kurmayer

**Affiliations:** 1 Research Department for Limnology, University of Innsbruck, Mondseestrasse 9, 5310 Mondsee, Austria; bmucholwa@gmail.com; 2 Department of Biological Sciences, Egerton University, P.O. Box 536, Egerton 20115, Kenya; soduor@egerton.ac.ke; 3 Environmental Sciences, Norwegian University of Life Sciences (NMBU), 1430 As, Norway; thomas.rohrlack@nmbu.no; 4 Department of Geosciences and the Environment, The Technical University of Kenya, P.O. Box 52428, Nairobi 00200, Kenya; sitoki@hotmail.com

**Keywords:** eutrophication, *Microcystis*, spatial variability, cyanotoxins, ELISA, PPIA, food chain, *Rastrineobola argentea*

## Abstract

The human health risks posed by exposure to cyanobacterial toxins such as microcystin (MC) through water and fish consumption remain poorly described. During the last two decades, coastal regions of Lake Victoria such as Nyanza Gulf (Kisumu Bay) have shown severe signs of eutrophication with blooms formed by *Microcystis* producing MC. In this study, the spatial variability in MC concentration in Kisumu Bay was investigated which was mostly caused by *Microcystis* buoyancy and wind drifting. Small fish (<6 cm) mainly composed of *Rastrineobola argentea* were examined for MC content by means of biological methods such as ELISA and protein phosphatase inhibition assay (PPIA) and partly by chemical-analytical methods such as LC-MS/MS. Overall, the MC content in small fish was related to the MC content observed in the seston. When comparing the MC content in the seston in relation to dry weight with the MC content in small fish the latter was found three orders of magnitude decreased. On average, the ELISA-determined MC contents exceeded the PPIA-determined MC contents by a factor of 8.2 ± 0.5 (SE) while the MC contents as determined by LC-MS/MS were close to the detection limit. Using PPIA, the MC content varied from 25–109 (mean 62 ± 7) ng/g fish dry weight in Kisumu Bay vs. 14 ± 0.8 ng MC/g in the more open water of L. Victoria at Rusinga channel. Drying the fish under the sun showed little effect on MC content, although increased humidity might indirectly favor photocatalyzed MC degradation.

## 1. Introduction

Lake Victoria has experienced major deterioration in its water quality mainly due to pollution and the introduction of exotic species [1]. The increased eutrophication is associated with urbanization, agricultural malpractices and deforestation [2]. Nutrients enter the lake from both point sources [1] and diffuse sources, including atmospheric deposition [3], which has exerted a considerable impact on the near shore areas [2,4].

Nyanza Gulf is one of the bays of Lake Victoria that is most affected by nutrient enrichment [5] which is coming from the highly populated catchment with mostly subsistence agriculture [2,6]. This has led to regular occurrence of bloom-forming cyanobacteria [7]. Some cyanobacterial species have the potential to produce cyanotoxins that pose a health risk to livestock and humans who rely on such water for drinking, sanitation, or as a food source [8]. The regular occurrence of cyanobacterial blooms in the Nyanza Gulf has been associated with fish kills and temporary shutdown of drinking water supply, i.e., from January to March 2004 [9].

The harmful cyanobacteria form surface scums due to a combination of buoyancy in the cells and wind action [10]. The buoyancy is caused by intracellular gas vesicles that decrease the specific density of the cells and is enhanced by cells forming large aggregates known as colonies. The diameter of the colonies can be several millimeters resulting in a rising of the cells up to the calm water surface within minutes [11]. These scums are also accumulated by wind action at the water surface which may result in the accumulation of cyanotoxins several orders of magnitude higher compared with the depth-integrated average concentrations [10]. The most common cyanotoxins are the hepatotoxic microcystins (MC) produced by cyanobacteria such as *Microcystis*, *Dolichospermum* (*Anabaena*), *Planktothrix* (*Oscillatoria*) and *Nostoc* [8]. The toxicity of MC is based on the potent inhibition of the protein phosphatases, PP1 and PP2a, disturbing cytoskeleton formation in eukaryotic cells. The oral LD_50_ for MC-LR in rats is 5 mg per kg of body weight that is comparable to the toxicity of cyanide [12]. Chronic MC exposure may promote tumor formation and has been linked to cancer development [13]. The colony-forming cyanobacteria such as *Microcystis* and *Dolichospermum* are most frequent in the Lake Victoria region and *Microcystis* is most frequently occurring in turbid and eutrophic water bodies [14]. It was shown that in Ugandan freshwater systems, the genus *Microcystis* rather than *Dolichospermum* or other genera produce MC [15]. 

MC is transferred through feeding from the cyanobacteria to zooplankton, fish and other aquatic biota and even to higher trophic levels [16,17,18]. Phytoplanktivouros fish such as filter-feeding *Oreochromis niloticus* are exposed to MC by unselective ingestion of toxic cyanobacterial cells [19]. In contrast, zooplanktivorous and carnivorous fish show a more direct feeding mode and could be exposed to cyanotoxins via trophic transfer [20]. On the other hand, even juvenile *Oreochromis niloticus* and smaller fish feeding more selectively on zooplankton in Murchison Bay, Lake Victoria, were observed to ingest toxic cyanobacteria along with detritus [21]. The occurrence of MC in fish tissue sampled from East African Lakes (including Lake Mburo, Napoleon Gulf and Murchison Bay of L. Victoria) has been demonstrated previously [19,22,23]. In order to assess human exposure risk, Poste et al. [23] pointed out the need to consider potential exposure to MC through fish consumption. These authors tested a variety of different fish species differing in feeding modes, including filter feeders, carnivorous feeders and small (juvenile) zooplanktivorous fish species. In contrast to large fish species where dorso-lateral fish muscle tissue was analyzed, the small fish (<10 cm) were analyzed in total. In this study, [23] the total MC concentrations were estimated by ELISA sometimes exceeding the Total Daily Intake (TDI) guideline (0.04 µg/kg body weight assuming a consumption of 100 g of fish daily) several fold [24]. The highest MC concentrations were determined in small (juvenile) zooplanktivorous fish such as *Haplochromis* spp. and the silver cyprinid *Rastrineobola argentea*, which could have resulted from the inclusion of toxic cyanobacteria in the gut. Commercial fishing for *R. argentea* started in the 1980s and today is considered the most important fishery by mass in the Lake Victoria region [25]. Small fish such as *R. argentea* are popular food around Lake Victoria and are marketed locally by women providing a cheap source of animal protein for the undernourished population [26,27]. These small fish are caught during the night using light traps and dried in the sun between 6 and 8 h the following day. Since the small fish are eaten as a whole, a transfer of MC to humans seems more likely when compared with fish muscle tissue consumption. It is known that MC cannot be degraded by sunlight alone. Indeed, the rate of MC degradation in the light depends on several factors such as wavelength (UV), pH and the presence of pigments or photocatalysts [12]. Because the small fish are dried in the sun for several hours, the exposure to sunlight might enable a breakdown of MC because ingested and lysing cyanobacterial cells in the gut would release photopigments as well. This study aimed to (i) quantify MC content in the locally consumed small fish in relation to the MC concentration in phytoplankton, and (ii) establish whether the processing of fish through drying in the sunlight has an effect on the final MC content in the fish. Fish sampled from inside the eutrophic Nyanza Gulf (ST1) near Kisumu were compared with fish sampled from Rusinga Channel located close to the main basin (ST2), that is less affected by eutrophication and MC contamination [9].

## 2. Results

### 2.1. Spatial Variation of Physical-Chemical Parameters

Over the entire study period, at ST1 (near Kisumu) the lake water was found to be rather turbid with low transparency (<26 cm). In contrast, at ST2 (close to the main basin) higher water transparency was recorded (85 cm). At ST1 higher conductivity was recorded while temperature and pH showed no variation between ST1 and ST2 (Table 1). For chlorophyll *a*, relatively low concentrations were recorded from the depth-integrated water samples both at ST1 (10–30 µg/L) and at ST2 in the main basin. Higher concentrations occurred at ST1 at the surface (19–50 µg/L), while maximum concentrations were recorded from the patches (274–4382 µg/L) forming bands possibly formed by Langmuir movements (Supplementary Figure S1). High chlorophyll *a* concentrations were also observed at the shore (18–1737 µg/L).

### 2.2. Phytoplankton Composition

Phytoplankton composition was dominated by Cyanobacteria, Bacillariophyceae, Cryptophyceae, and Chlorophyceae; however, all groups occurred with relatively few taxa only. Cyanobacteria were composed mainly of the genera *Microcystis*, *Planktolyngbya* and *Dolichospermum* (*Anabaena*) at both ST1 and ST2. The Bacillariophyceae, Cryptophytes and Chlorophytes were dominated by *Nitzschia*, *Cryptomonas* and *Chlamydomonas*, respectively (Supplementary Table S1).

**Figure 1:**
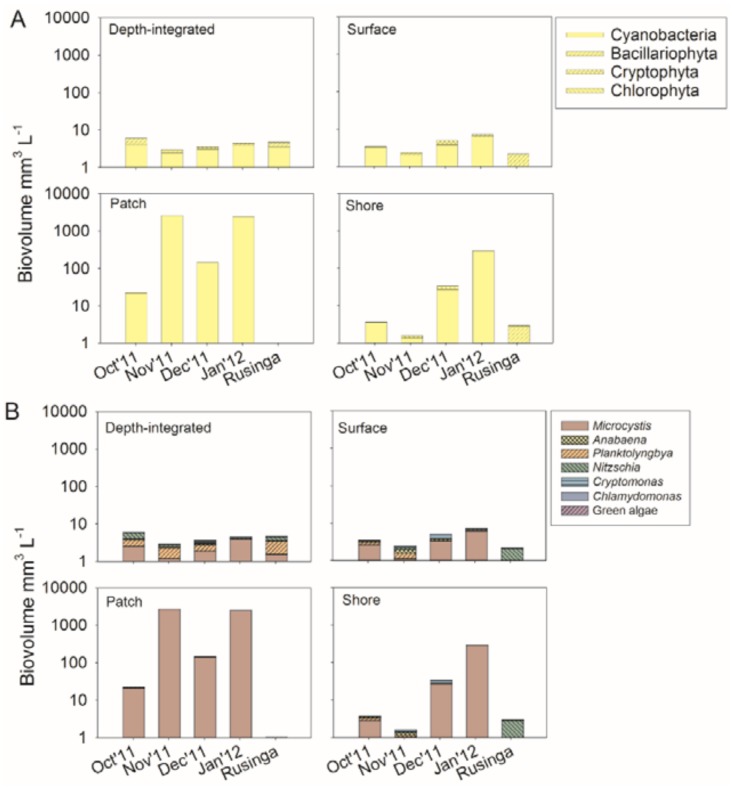
Phytoplankton biovolume composition from different sample types (depth-integrated, surface, patch and shore) in Nyanza Gulf and Rusinga Channel during October 2011 to January 2012. (**A**) phytoplankton groups (relative proportion >5%), (**B**) phytoplankton genera (relative proportion >5%).

The cyanobacteria dominated in all depth-integrated samples at ST1 and ST2. Typically, this dominance was due to the abundance of *Microcystis* (54 ± (SE) 10%) and *Planktolyngbya* (20 ± 8%). When compared with depth-integrated samples, the proportion of *Microcystis* increased significantly in patch (98 ± 2%) and shore (80 ± 7%) samples (Repeated Measures ANOVA on Ranks, *p* < 0.001, Tukey test for post-hoc pairwise multiple comparison, *p* < 0.05). In contrast, a decrease in proportion of *Planktolyngbya* in patch (1 ± 1%) and shore (5 ± 3%) samples was found (Repeated Measures ANOVA on Ranks, *p* ≤ 0.001, Tukey test *p* < 0.05). The patch and shore samples also showed maximum phytoplankton biovolume that differed from depth-integrated samples on average by an order of magnitude (Figure 1). In contrast, the changes in phytoplankton composition at the surface of the water column were less visible. The Bacillariophyceae contributed the second most abundant algal class, most importantly by the genus *Nitzschia*. The proportion of *Nitzschia* decreased when comparing depth-integrated (13 ± 6%) samples with patch (1 ± 1%) and shore (2 ± 1%) samples (Repeated Measures ANOVA on Ranks, *p* = 0.002, Tukey test *p* < 0.05). In summary, the general dominance of *Microcystis* in phytoplankton became even more pronounced among patch and shore samples. There was a positive correlation between the two phytoplankton biomass estimates, phytoplankton biovolume and chlorophyll *a* concentrations (R^2^ = 0.81, *p* < 0.001), (Supplementary Figure S2).

### 2.3. Microcystin Concentrations in Water

MC was detected in all water sample types collected from Kisumu Bay (*n* = 20) but not in any samples from Rusinga channel. Four MC structural variants occurred: MC-YR (*m*/*z* = 1045 with retention time of 17 min), MC-LR (*m*/*z* = 995, 18 min) and two unknown MC variants eluting at 24 min (*m*/*z* = 1052) and 25 min (*m*/*z* = 1002), respectively. On average, MC-YR and MC-LR and MC *m*/*z* 1052 accounted for the major part of the total MC (Table 2). The proportion of MC-YR was significantly higher and that of MC *m*/*z* 1052 was lower in patch samples when compared with depth-integrated samples. 

**Figure 2:**
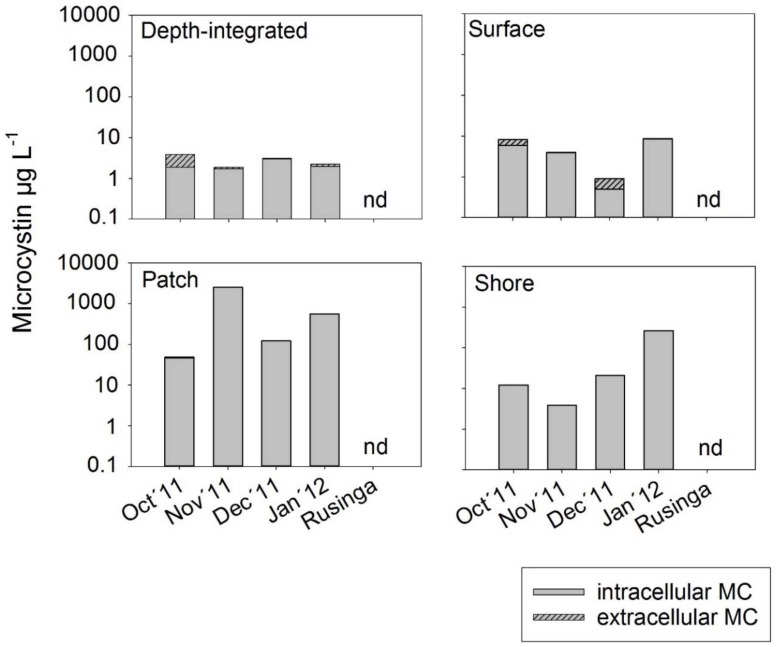
Intracellular microcystin concentrations (in MC-LR equivalents) as determined by HPLC-DAD and dissolved MC concentrations as determined by ELISA from different sample types in Nyanza Gulf and Rusinga Channel during October 2011 to January 2012. (n.d, not detected).

The highest total MC concentrations were recorded in patch samples with maximum concentrations exceeding 2 mg/L of MC-LR equiv. (Figure 2) as recorded in November 2011. MC concentrations also increased in shore water samples exceeding 100 µg/L in January 2012. In contrast, total MC concentrations from depth-integrated and surface samples were below 10 µg/L. In general, the dissolved MC showed low concentrations only (<3.3 µg/L). On average, the patch samples had five times higher MC concentration than the depth-integrated sample, while the shore samples had MC concentrations twice that of the depth-integrated sample. For the cell-bound fractions, the MC concentrations were 387, 28 and 2 times higher in the patch, shore and surface water samples respectively when compared with the depth-integrated sample. The total MC concentrations were significantly related to *Microcystis* cell numbers (*n* = 19, R^2^ = 0.71, *p* < 0.0001) but not to any other abundant cyanobacteria (i.e., *Planktolyngbya*, *Anabaena*) (Supplementary Figure S3). This suggests that other genera than *Microcystis* potentially producing MC were not important during the study period. Average cellular MC contents varied from 2–250 fg (mean ± SE, 49 ± 9) MC per *Microcystis* cell, and did not differ between sample types (Repeated Measures ANOVA on Ranks, *p* = 0.139). On a dry weight (DW) basis the average cellular MC content varied from 247 and 261 µg MC/g DW in depth-integrated and surface samples to 495 µg MC/g DW in patch and shore samples but did not differ between sample types (Repeated Measures ANOVA on Ranks, *p* = 0.65). Surprisingly, intracellular MC and dissolved MC concentrations were not related. For example, the highest intracellular MC concentrations in patch samples had only 0.4–3 µg/L of dissolved MC. This discrepancy might be explained by high turbulence induced by wind action leading to a constant dilution of dissolved MC that, however, was overcome by the buoyant *Microcystis* colonies forming the patches.

### 2.4. Small Fish Species Composition

The following small fish species were identified in Kisumu Bay (ST1): *Barbus* sp., *Haplochromis* sp., *Lates niloticus* and *R. argentea*. All samples were dominated by *R. argentea* both in terms of individual numbers (>55%) and in terms of fresh weight (>51%), (Table 3). In Rusinga channel (ST2) only three fish species were identified (*R. argentea*, *L. niloticus* and *Barbus* sp.) and *R. argentea* showed the maximum proportion (95%). 

During the study period, the average fresh weight of individual *Barbus* sp. was highest (mean ± SE, 1.0 g ± 0.07), while it was 0.6 g ± 0.02 for *R. argentea*, 0.6 g ± 0.07 for *L. niloticus* and 0.6 g ± 0.2 for *Haplochromis* sp. In the Rusinga channel, ST2 individuals of *L. niloticus* were heavier (1.8 g ± 0.2), while *R. argentea* was on average lighter (0.5 g ± 0.02). 

In Kisumu Bay, *Barbus* sp. had the highest total length (47 ± 1.3 mm) while *R. argentea* (39 ± 0.6 mm), *L. niloticus* (35 ± 1 mm) and *Haplochromis* sp. (32 ± 3 mm) were on average smaller. In Rusinga, *L. niloticus* was longer (58 ± 2 mm), while *R. argentea* (40 ± 0.5 mm) did not show a difference in length. As prey size selection depends to a significant extent on fish size, it is concluded from the observed length distribution that the sampled fish were mostly feeding on plankton.

### 2.5. Microcystin Content in Small Fish as Determined by Biological and Chemical-Analytical Methods

**Figure 3:**
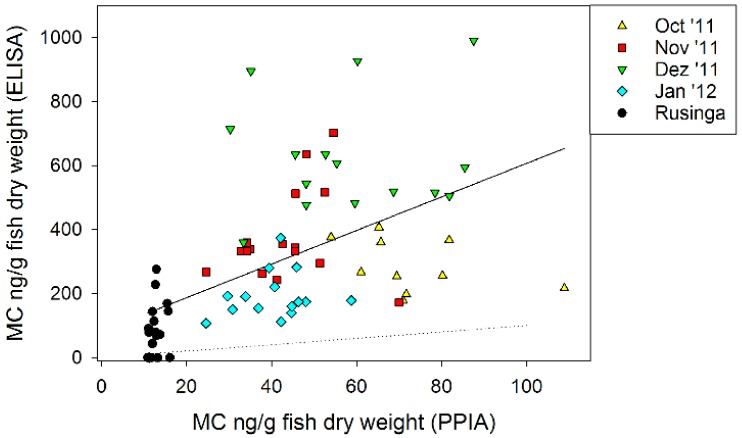
Correlation of microcystin contents in small fish (ng/g in dry weight) as determined by ELISA vs. microcystin contents as determined by the PPIA (R^2^ = 0.29, *p* < 0.0001). The PPIA results were on average 8.2 (±0.5)-fold lower than those obtained by ELISA. The one-to-one relationship is indicated by the dotted line.

MC occurred in fish samples obtained from Kisumu Bay (ST1) and Rusinga channel (ST2), as revealed by both biological methods, ELISA and PPIA. The MC contents ranged from 0–990 and 11–109 ng MC/g DW for ELISA and PPIA, respectively. Altogether the MC contents as determined in fish by ELISA were linearly related to the MC contents as determined by PPIA: y = 82.2 + 5.2x, where y is MC in ng/g DW as determined by ELISA and x is MC in ng/g DW as determined by PPIA (R^2^ = 0.29, *p* < 0.0001). On average, the ELISA-determined MC contents showed a higher variability and exceeded the PPIA-determined MC contents by a factor of 8.2 ± 0.5 (Figure 3). 

All samples obtained from December 2011 were additionally tested for MC content using chemical-analytical methods such as LC-MS/MS (*n* = 16). In four samples, MC-YR was detected ranging from 8–20 ng MC/g DW while the other MC variants were not observed. It is concluded that MC-YR indeed specifically occurred in small fish. However, compared with water samples (i.e., µg of MC per g DW of plankton biomass as determined by HPLC) the concentration of MC-YR in small fish tissue was found to be very low (i.e., ng of MC per g DW of fish biomass as determined by LC-MS/MS). 

### 2.6. Microcystin Content in Small Fish in Relation to MC in Phytoplankton

**Figure 4:**
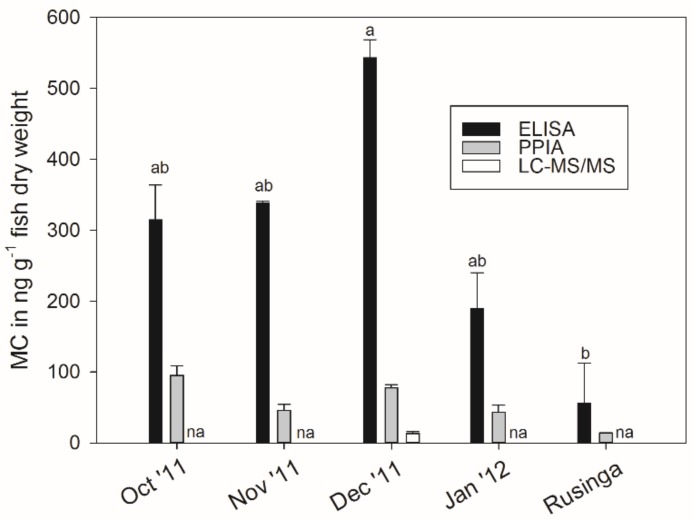
Mean (±SE) microcystin contents in small fish (in ng/g dry weight) as determined by ELISA and PPIA and LC-MS/MS originating from Kisumu Bay in Nyanza Gulf and Rusinga Channel during October 2011 to January 2012 (n.a, not analyzed). Letters indicate subgroups not significantly different at *p* < 0.05 if an overall difference was found (Friedman Repeated Measures ANOVA on Ranks).

Over the study period, fish samples originating from Kisumu Bay (ST1) had a higher MC content when compared with fish samples obtained from Rusinga channel (ST2), i.e., MC contents ranged from 190 ± 51 to 543 ± 26 ng MC/g DW in Kisumu Bay vs. 56 ± 56 ng MC/g DW in Rusinga channel as determined by ELISA (Repeated Measures ANOVA on Ranks, *p* = 0.034, Tukey test *p* < 0.05). Analogously, MC content varied from 43 ± 10 to 95 ± 14 ng MC/g DW in Kisumu Bay vs. 14 ± 0.8 ng MC/g DW in Rusinga channel as determined by PPIA (Repeated Measures ANOVA on Ranks, *p* = 0.107), (Figure 4). Thus the MC content in fish was related to the MC content in the seston, i.e., higher MC contents in fish from Kisumu Bay vs. lower MC contents in fish sampled from more open water at Rusinga channel. Using both biological methods the MC content in fish was found decreased by a factor of 10^3^ implying significant biodilution rather than biomagnification through the food chain.

**Figure 5:**
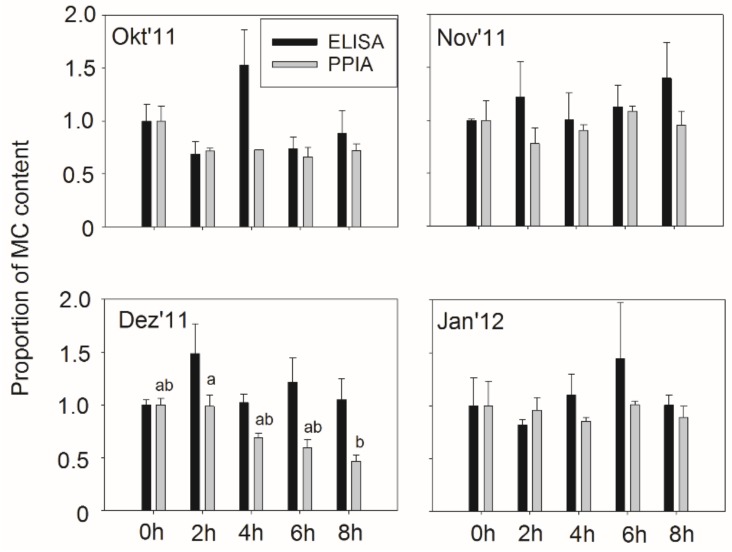
Mean (±SE) proportion of microcystin contents in small fish after 0–8 h of sun drying. Letters indicate subgroups not significantly different at *p* < 0.05 if an overall difference was found (Friedman Repeated Measures ANOVA on Ranks).

In order to find out whether the air-drying time influenced the MC content in small fish, the proportion of MC content in relation to t_0_ was calculated. There was no clear trend in MC content in relation to MC content at t_0_ for all sampling dates as inferred from the ELISA. However, there was a decrease in MC content as determined from PPIA in relation to t_0_ with an increase in exposure time (2–8 h) in October and December samples (Figure 5). During the December experiment, the average MC content 77 ± 5 ng MC/g DW significantly decreased down to 36 ± 5 ng MC/g DW (Repeated Measures ANOVA on Ranks, *p* < 0.027). During October, the average MC content 95 ± 14 ng MC/g DW also decreased down to 69 ± 6 ng MC/g DW (Repeated Measures ANOVA on Ranks, *p* = 0.147). The recording of meteorological data at Kisumu Airport showed relatively low variability in daily temperature (22.7–25 °C) and irradiance (866–1387 µmol photons. m^−2^·s^−1^, Supplementary Table S2). In contrast, the relative humidity varied and showed maxima of 80% in October and December while it was lower in November (35%) and in January 2012 (62–66%), (Supplementary Table S2). In summary, the MC content in small fish showed a relatively high stability during the drying process and the overall relation to MC content in seston was not changed.

## 3. Discussion

### 3.1. Eutrophication and Spatial Variability of Phytoplankton Composition

The Nyanza Gulf is a semi-closed bay, therefore, it has a limited exchange of water with the main basin which is related to increased turbidity due to high algal biomass [28,29]. In other bays of Lake Victoria and open waters such as the Rusinga channel, there is dilution because of water exchange with the main basin, which can explain the lower phytoplankton biovolume [4]. Besides algal growth, the suspended solids from three river inflows in this area (Sondu, Kisat and Nyamasaria) also further increase the turbidity, i.e., satellite images show sediment-laden inflow waters during the rainy seasons [29]. The high turbidity reduces light availability and selectively favors specialized algal species such as *Microcystis* capable of remaining near the water surface through the presence of gas vesicles [30]. Particularly in shallow aquatic ecosystems, buoyant cyanobacteria of the genus *Microcystis* are favored by both eutrophication and turbidity and have been found to be dominant in freshwater ecosystems which are naturally eutrophic (such as Lake George in Uganda) [14]. The larger size of the buoyant colonies not only enables pronounced vertical migration but also accumulation within Langmuir circulation where positively buoyant particles will accumulate above the downwelling region [31]. In contrast, nonbuoyant phytoplankton such as cyanobacteria *Planktolyngbya* and diatoms of the genus *Nitzschia* were unaffected when compared with depth-integrated samples and thus reduced in proportion. For another large lake, Lake Biwa in Japan, Ishikawa et al. [31] further described large circular horizontal currents that accumulate *Microcystis* in the center transporting *Microcystis* from inshore to offshore areas. Indeed, for the eastern part of the Nyanza Gulf a large circular current has been described which contributes to the strong horizontal gradient in salinity from the eastern part to the western part towards the main basin [29]. The larger scale hydrodynamic forces may help in supporting the more stable formation of Langmuir circulations which depend on moderate wind speed and the presence of wind waves [31]. In general, the pronounced physico-chemical gradient from the eastern part to the western part of Nyanza Gulf has been increased by reducing the hydrological connectivity to the main basin [31]. Most importantly, a causeway was constructed to link Rusinga island with the main land in 1983, which reduced the water exchange with the main basin [32]. However, in May 2017, the Mbita channel was re-opened to reconnect Nyanza Gulf with the main Lake Victoria besides Rusinga channel. This opening (now called Mbita channel) with a width of 150 m is supposed to increase ecological and hydrological connectivity and might also reduce the internal physical stability of the eastern part of Nyanza Gulf. 

### 3.2. Spatial Variability of Microcystin Concentration in Water

Spatial and temporal variability in MC concentration has been described along the shoreline of wind-exposed systems worldwide and has been considered as an important variable in estimating health risks through exposure to toxic algal blooms. While maximum MC concentrations are typically observed along the shore [10], in this study, maximum MC concentrations occurred within the patches drifting at the surface in Kisumu Bay (Supplementary Figure S1). The positive correlation between MC concentration and *Microcystis* cell numbers showed that *Microcystis* sp. was the major MC producer while the contribution of other cyanobacteria species was of minor importance. This is in agreement with earlier results from Uganda and Nyanza Gulf, respectively [9,15]. Thus the lack of detectable MC in all sample types obtained from Rusinga channel can be best explained by the general lowest numbers of *Microcystis* (<100 cells/mL). In contrast, in the eutrophic Nyanza Gulf, the selective entrainment of *Microcystis* in the Langmuir circuits as described above increased the horizontal patchiness in MC concentration considerably. When compared with depth-integrated samples, the proportions of the more abundant MC structural variants changed, i.e., the proportion of MC-YR increased and the unknown MC *m*/*z* 1052 variant decreased (Table 2). This change in MC-variant proportion might imply a change in genotype composition of *Microcystis* sp., for example, through favoring the large sized colonies such as *M. aeruginosa* vs. other morphospecies that occur as smaller sized colonies [33]. The genus *Microcystis* shows impressive variability of cell numbers per colony ranging from a few cells to >10^5^ cells, which can be explained in part by the variable cationic chemical composition of the mucilage embedding the cells [34]. Notably, the large sized *Microcystis* colonies frequently show maximum MC contents while smaller sized *Microcystis* colonies typically show the lowest MC contents, i.e., as reported from Wannsee in northern Germany [33], from northern Missouri and Iowa in North America [35] and Lake Taihu in China [36]. Thus, the rather high MC concentrations observed in this study within the patches may be further increased by selective enrichment of large sized buoyant *Microcystis* colonies.

### 3.3. Microcystin Content in Small Fish as Determined by Biological and Chemical-Analytical Methods

The extraction and detection of MC from complex matrices such as fish tissue is an important question and both free and covalently-bound MC need to be distinguished [37]. Methods measuring free MC may underestimate the total MC content because of covalent binding of the Methyl-dehydro-alanine (Mdha) residue in pos. 7 of the MC molecule to the active site of PP or other cysteine-containing peptides such as glutathione. These covalently bound MC can be released by oxidation through the so-called Lemieux technique resulting in an oxidative derivative of the Adda side chain as 2-methyl-3-methoxy-4-phenylbutyric acid (MMPB) which is measured by LC-MS/MS [38]. It is under question whether the covalently bound MC is toxicologically active, i.e., through slow release by digestive enzymes during gut passage [39]. The proportion of covalently-bound MC in fish tissue can be substantial [20] (and references therein) and ELISA has been shown to be able to detect the cysteine-MC and the glutathione conjugates. Thus, it is not surprising that MC concentrations as estimated by ELISA were highest also in this study because of its crossreactivity with both detoxification and PP conjugates. 

In contrast to ELISA, the PPIA measures the effect of inhibitors such as MC on the activity of PP monitored through enzymatic release of a chromogenic substrate. Thus the PPIA quantifies the toxicity of a sample and yields toxicity equivalents. ELISA is based on structural recognition of MC molecules and is designed to yield toxin concentrations in MC-LR equivalents. As other natural products of cyanobacteria (e.g., anabaenopeptins, okadaic acid) also inhibit PP and MC structural variants differ in activity, the results obtained by the two biological methods cannot be directly compared. However, in general, PPIA is expected to target the free MC. 

On the other hand, chemical-analytical techniques such as LC-MS/MS are known to be highly specific with high resolution and are able to detect known MC molecule parent and fragment ions even in complex sample matrices [8]. However, because of the high target molecule specificity, covalently-bound MC also is likely overlooked. This target molecule specificity is less relevant in LC-DAD (UV based) methods, however, the latter are generally less sensitive and suffering from sample matrix effects [8]. In summary, it is rather the combination of biological and chemical-analytical methods that is used to evaluate the exposure risk through small fish consumption. In this study the occurrence of MC-YR in fish sample extracts was unambiguously identified while the MC contents estimated by ELISA might imply a significant share of bound MC through various catabolic processes. Since the actual toxicity is most directly addressed by the PPIA, in this study the PPIA-derived estimates have been used to estimate the exposure risk. 

### 3.4. Microcystin Content in Small Fish

The MC contents, as determined by ELISA in this study (190 ± 51 to 543 ± 26 ng MC/g DW), were in the range of MC contents reported previously by Poste et al. [23,40]. For example, *R. argentea* and *Haplochromis* sp. species sampled from Murchison Bay and Napoleon Gulf contained 36–41 and 39–129 µg/kg wet weight that would translate into 360–1290 µg/kg DW (assuming dry weight to be 10% of wet weight). The MC contents derived by ELISA were quantitatively supported by PPIA, i.e., small fish sampled from Rusinga channel contained significantly less MC as observed by both methods (Figure 3 and Figure 4). The general experience is that active MC have a proportion which is one order of magnitude lower when compared with the total extractable MC [20]. Indeed, in this study MC contents in small fish, as estimated by PPIA, were on average eight-fold lower when compared with ELISA-derived estimates. The World Health Organization (WHO) recommended the tolerable daily intake (TDI) for MC over the lifetime of a human being to be 0.04 μg MC-LR equivalents per kg of body weight per day [24]. Using this guideline, the tolerable dose of MC-LR is calculated assuming a consumption of 100 g (wet weight) of fish per day. In this study, the PPIA-derived estimate would translate to 246–519 ± 24–1089 ng of MC-LR equiv. (min–mean ± SE–max) consumed from small fish in Kisumu bay per day, which would be below the theoretical tolerable intake for an adult (2.4 µg for a 60 kg adult). 

When compared with muscle tissue, the higher MC contents can be explained by the inclusion of the viscera containing *Microcystis* colonies in the analyzed sample [20]. As small fish have been found to contain a significant amount of *Microcystis* in their diet (e.g., 80% of phytoplankton), this diet-based vector is considered a relevant one [21]. The surprisingly high proportion of phytoplankton (*Microcystis* sp.) in the diet of zooplanktivorous fish may be linked to the general high turbidity in the Gulf system but also has been reported from other systems (e.g., in Rwanda [41]). Currently, the diet composition and the possible relation to MC content in small fish along the eutrophication gradient in Nyanza Gulf is not known but should be explored further. The feeding behavior of small fish and a possible avoidance of the observed patches with *Microcystis* dominance and maximum MC concentrations would also be a research route. On the other hand, the lysed cyanobacteria in the gut actually release not only the MC but also photopigments such as phycobilins known to act as photo sensitizers accelerating the photolysis of MC [42]. Thus, exposure of the small fish containing phytoplankton in the diet in the sunlight actually might result in reduced MC contents. In this study, such significant reduction in MC content was only observed at relatively high humidity conditions (Figure 5, Supplementary Table S2). This result would imply that an accelerated drying process actually would reduce the photolysis potential because photocatalyzed degradation is occurring in the aquatic solution [43]. Nevertheless, the photolysis-induced MC degradation would be a relatively simple measure to reduce the MC content in the small fish, for example, by maintaining the humidity of the fish biomass during a certain time of sun exposure.

## 4. Conclusions

The eutrophication of the eastern part of Nyanza Gulf has led to high concentrations of MCs, which is mainly caused by the dominance of the bloom-forming cyanobacterium of the genus *Microcystis*. The buoyant *Microcystis* colonies accumulated in the patches through advective currents possibly formed by Langmuir circulations linked to maximum MC concentrations in the patches. In consequence, MC contents have been observed in small planktivorous fish species such as *R. argentea*. Although active MC in small fish was detected regularly, the theoretical lifetime tolerable daily intake for adults was not exceeded. The fish drying process in the sun did not consistently reduce the MC content in the fish which might be explained by the variable relative humidity. However, since photocatalyzed degradation of MC in the sunlight is a fast process, exposing the fish in wet conditions to the sun for a short time period might be a possibility to reduce MC content in food.

## 5. Materials and Methods

### 5.1. Study Area and Sampling

**Figure 6:**
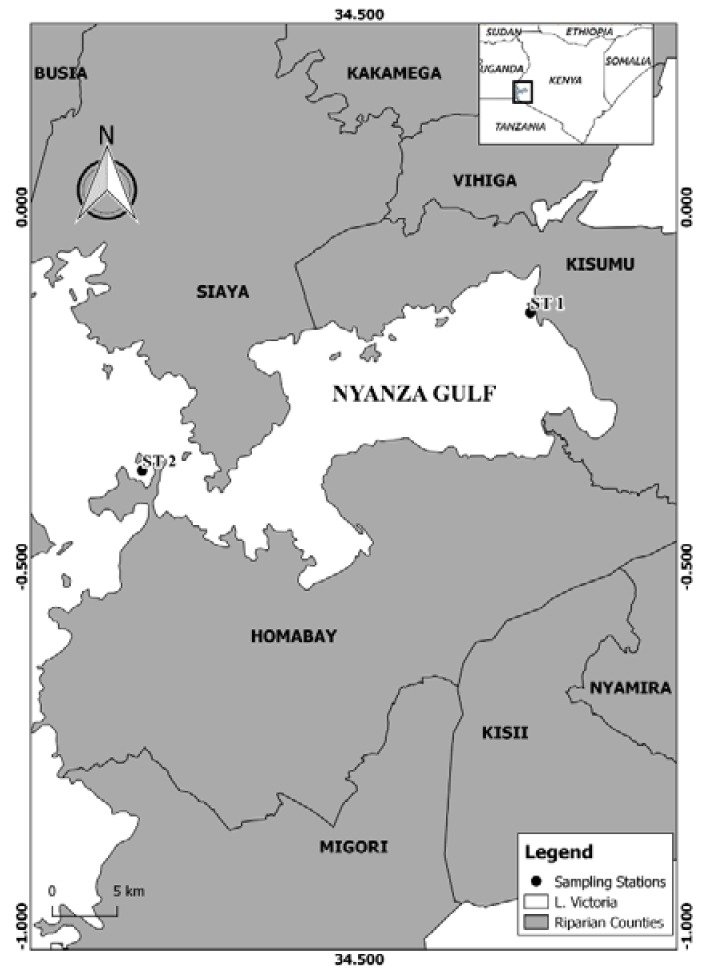
Map of Lake Victoria, Kenyan side, showing Nyanza Gulf and the two sampling stations (ST1, Kisumu Bay and ST2, Rusinga Channel).

Nyanza Gulf is located in the North-Eastern part of L. Victoria and is connected to the main basin via the Rusinga channel. Besides Mwanza Gulf (Tanzania) and Napoleon Gulf (Uganda), Nyanza Gulf is one of the largest bays of Lake Victoria with an area of 1400 km^2^. With an average depth of 5 m, it is relatively shallow in comparison with the main basin [28]. Samples were collected monthly (from October 2011 to January 2012) at one sampling station in eutrophic Kisumu Bay (ST1: S 00°10′26.7′′ E 34°44′11.7′′) and once in January 2012 in Rusinga Channel (ST2: S 00°22′46.2′′ E 34°11′15.0′′), (Figure 6). Water temperature, dissolved oxygen, pH and conductivity were determined at 1-m depth using a multiprobe (Hach-Lange, Düsseldorf, Germany). 

Depth-integrated water samples were obtained by mixing 1 liter collected from every meter through the water column down to 3 m depth using a Van Dorn sampler. Surface samples were taken from scums visible as patches possibly formed by Langmuir spirals (“patch” samples) or at the shore (“shore” samples). Water samples were filtered using Whatman GF/C filters. For chlorophyll *a* analysis, the filters were stored frozen and processed the following day. For cell bound MC analysis, filters were dried overnight (50 °C) and stored frozen. The filtrate was used for determination of dissolved MC in water using ELISA.

### 5.2. Phytoplankton Composition and Abundance

Chlorophyll *a* was extracted using hot ethanol [44]. Phytoplankton composition was determined from 2 mL of Lugol-fixed samples using an inverted microscope at 400× magnification [45]. Phytoplankton taxa were identified from morphological characteristics according to Talling, Komàrek and Kling, Cronberg and Anadotter [46,47,48]. In general, phytoplankton specimen were counted as distinct cells (*Microcystis* sp., *Anabaena (Dolichospermum)* sp., *Merismopedia* sp.; or the pennate diatom *Nitzschia* sp.). Filamentous cyanobacteria were counted as filaments (*Planktolyngbya* sp.). In order to determine phytoplankton biovolume of individual taxa the biovolume was determined by geometric approximation [45]. To quantify *Microcystis* cells the colonies were disintegrated into single cells using sonication for 10 s (output 40 watt) by a sonicator (Heat Systems-Ultrasonics, Inc., Plainview, NY, USA, 11,803) [49]. Pilot experiments revealed that maximum *Microcystis* cell numbers were obtained after 10 sonication treatments (in 10 mL volume).

### 5.3. Microcystin Determination in Water

The dissolved MC in water was determined directly in the filtrate using indirect competitive ELISA (Abraxis LLC, Warminster, PA, USA, Microcystins-ADDA ELISA kit, PN 520011) according to the manufacturer’s instructions and reading absorbance was calibrated though the provided MC-LR standards at 450 nm wavelength. The ELISA has a limit of detection of 0.1 ng/mL (0.1 µg/L). For the cell-bound fraction of MC, cells were collected on filters and biomass was extracted in aqueous methanol (75%, *w*/*v*) according to Fastner et al. [50]. MC structural variants were chromatographically separated using High performance liquid chromatography with diode array detection (HPLC-DAD) as described [33]. MC variants were identified by their characteristic absorption spectra and retention times [15] and quantified at 240 nm wavelength. Using MC-LR as the external standard, the concentration of MC-LR equivalents was calculated from the regression curve y = 1885.3x − 6.8775, (R^2^ = 0.99), where (y) was the absorption (mAU) recorded at 240 nm and x represented the injected concentration (ng) of MC-LR standard (CyanoBiotech GmbH, Berlin, Germany). Under the specified conditions, 50 ng of MC-LR equivalents injected could be resolved resulting in a theoretical limit of detection of 300 ng of MC-LR (=0.6 µg/L assuming a typical filtration volume of 500 mL).

### 5.4. Fish Sampling

**Figure 7:**
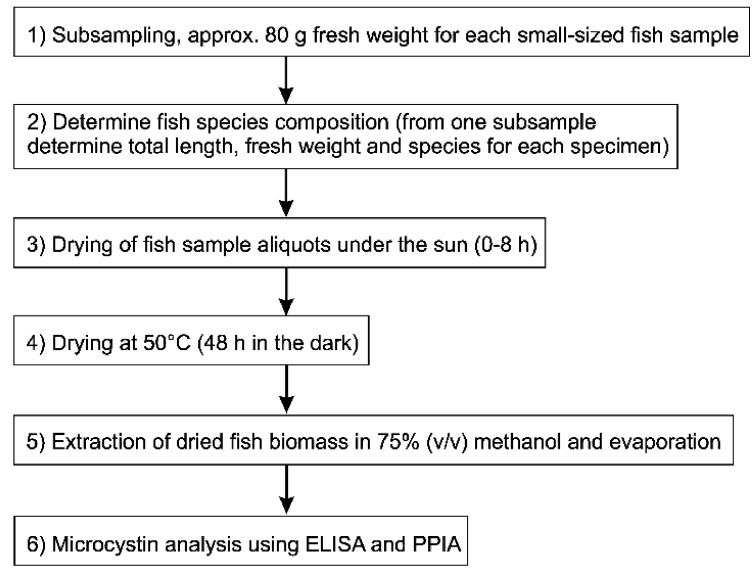
The workflow for fish sample processing including sampling, drying, extraction and MC analysis.

Fishing for small fish (mostly *R. argentea*) was performed using light attraction by means of kerosene pressure lamps floating on the water surface during moonless nights. Samples were obtained monthly from fishermen early in the morning from Kisumu Bay (ST1) during October 2011–January 2012 as well as Rusinga channel (ST2) in January 2012. Fish samples were packed immediately on ice and were transported to the laboratory for further processing within 12 h. Fish samples were subsampled, taking aliquots of approximately 80 g wet weight at random. The species composition, fresh weight (g) and total length (mm) for each specimen from one aliquot subsample were determined. Fish samples were dried either directly (50 °C) or dried under the sun for 2, 4, 6 and 8 hours respectively, and subsequently dried at 50 °C (Figure 7, Supplementary Figure S4). Individual fish sample aliquots had a dry weight of 18.3 ± 0.6 (mean ± SE), 16.8 ± 0.2, 16.6 ± 0.3, 16.9 ± 0.2, 17.1 ± 0.2 g in October, November, December, and January at ST1 and in January at ST2, respectively.

### 5.5. Microcystin Analysis in Small Fish

MC extraction from dried fish samples was done by aqueous methanol as described [51]. The extraction process was repeated three times. In the first two extraction steps, the dried and weighed fishes were ground as a whole in a mortar and extracted in 75% (*v*/*v*) methanol (2 h, 4 °C). After centrifugation the pellet was resuspended in 30 mL of 75% (*v*/*v*) methanol containing 0.002% (*v*/*v*) glacial acetic acid (24 h, 4 °C). The next day the pooled supernatant was centrifuged to remove particles and the cleared extract was evaporated to dryness (BUCHI Rota vapor R-205) and stored at −20 °C. The residues from the evaporation were resuspended in 2 mL of methanol and transferred to glass bottles kept open under the fumehood overnight to evaporate the methanol (as methanol is known to interfere with ELISA, [52]). To enhance the evaporation of methanol the extract was incubated at 60 °C for another 2 h. The extract was then diluted with distilled water (1:1000) in glass vials and MCYST concentration was determined by the same indirect competitive ELISA (Microcystins-ADDA ELISA kit, PN 520011) as described above. Using the same MC-LR standard, the concentrations were expressed as MC-LR equivalents in nanograms per gram of fish dry weight. In order to identify the proportion of active MC in the fish tissues the standard protein phosphatase inhibition assay (PPIA) technique [53] was used for aliquots of the fish extract samples dissolved in methanol and stored at −20 °C. A commercially available microplate test kit (Microcystest) from Zeulab (Zaragoza, Spain) has been used and 250-fold dilutions of fish sample extracts were analyzed according to the manufacturer’s instructions. The inhibition of the PP2A activity was monitored using the conversion of chromogenic substrate and reading absorbance at 405 nm wavelength. Finally, using all fish samples from December 2011 the MC was quantified using chemical-analytical methods by LC-MS/MS [19]. The tandem mass-spectrometry instrumental setup included a water ACQUITY UPLC system directly connected to a Quattro Premier XE MS/MS detector. MC structural variants were quantified in positive ESI mode using multiple reaction monitoring according to the retention time and the precursor ion. The daughter ion monitored for all MCs was the typical 135 Da Adda fragment. All MS settings were optimized with commercial standards (purchased from Sigma, Oslo, Norway) which were also used to calibrate the system: MC-RR *m*/*z* = 519.8 [M + 2H]^2+^, MC-YR *m*/*z* = 1045.5 [M + H], MC-LR *m*/*z* = 995.5 [M + H]. The detection limit was 40 ng/mL for MC-YR and MC-LR (10 µL injection volume).

### 5.6. Statistical Analysis

Phytoplankton composition and MC contents were compared between samples using the Friedman repeated measures analysis of variance (ANOVA) on ranks (*p* < 0.05) followed by pairwise multiple comparison (Tukey test, *p* < 0.05) using SigmaPlot (Version 13.0, Systat Software Inc, San Jose, CA, USA).

## Figures and Tables

**Table 1:** Physicochemical and biological variables (mean ± SE) during the study period (October 2011 to January 2012).

Date	Temp. (°C)	Cond. (µS/cm)	pH	Secchi (cm)	Chlorophyll *a* (µg/L)				Total Phytoplankton Biovolume (mm^3^/L)			
					Surface	Integrated	Patch	Shore	Surface	Integrated	Patch	Shore
Kisumu Bay (ST1)												
27 October 2011	25.3 ± 0.1	153 ± 0.4	6.5 ± 0.3	25	50	14	1038	128	3.5	3.9	22.4	3.7
8 November 2011	n.d	159 ± 0.1	6.3 ± 0.2	15	20	17	274	18	2.4	2.1	264	1.6
5 December 2011	24.9 ± 0.3	130 ± 0.3	6.5 ± 0.1	27	19	10	845	73	5.0	3.0	145	34
10 January 2012	25.5 ± 0.2	134 ± 0.3	n.d	7	44	30	4382	1737	7.3	4.5	2467	291
**Rusinga Channel (ST2)**												
9 January 2012	25.8 ± 0.1	106 ± 0.1	n.d	85	13	14	n/a	15	2.2	2.6	n/a	3.0

n.d not determined; n/a, not applicable.

**Table 2:** Average (±SE) proportion of microcystin structural variants and microcystin concentrations (in MC-LR equiv.) recorded at Kisumu Bay from various sample types (*n* = 4).

Structural Variant	Retention (min)	Surface	Integrated	Patch	Shore	*p*-Value ^1^
Proportion						
MC-YR	17.06–17.26	40 ± 5 ^a,b^	26 ± 6 ^b^	45 ± 2 ^a^	33 ± 4 ^a,b^	0.015
MC-LR	18.34–18.52	28 ± 4	18 ± 4	29 ± 2	26 ± 4	0.136
MC *m*/*z* 1052	23.95–24.16	25 ± 9	51 ± 12	16 ± 1	31 ± 9	0.05
MC *m*/*z* 1002	25.0–25.07	7 ± 3	6 ± 2	11 ± 2	9 ± 2	0.463
**Concentration (µg/L)**						
MC-YR	17.06–17.26	0.6 ± 0.4 ^a,b^	0.5 ± 0.1 ^b^	343 ± 186 ^a^	29 ± 17 ^a,b^	<0.001
MC-LR	18.34–18.52	1.3 ± 0.3 ^b^	0.4 ± 0.1 ^b^	210 ± 102 ^a^	22 ± 12 ^a,b^	<0.001
MC *m*/*z* 1052	23.95–24.16	1.4 ± 0.6 ^b^	1.2 ± 0.3 ^b^	160 ± 94 ^a^	17 ± 9 ^a,b^	<0.001
MC *m*/*z* 1002	25.0–25.07	0.6 ± 0.5 ^b^	0.2 ± 0.04 ^b^	111 ± 60 ^a^	9 ± 5 ^a,b^	<0.001

^1^ Friedman Repeated Measures ANOVA on Ranks (Superscripts indicate homogeneous subsets not significantly different at *p* = 0.05 according to Tukey-test post-hoc pairwise comparison).

**Table 3:** Number (proportion) of individuals assigned to small fish species sampled in Nyanza Gulf (ST1) during October 2011–January 2012 and in Rusinga channel (ST2) in January 2012, as well as average ± SE (min–max) fresh weight and total length.

	*Barbus* sp.	*Haplochromis* sp.	*L. niloticus*	*R. argentea*
Number (Proportion)				
ST1, October 2011	26 (33)	2 (2.6)	6 (7.8)	43 (55.5)
ST1, November 2011	17 (10)	2 (2.2)	51 (30.5)	97 (58)
ST1, December 2011	3 (2.5)	0	44 (37.3)	71 (60.2)
ST1, January 2012	19 (13)	0	18 (12.6)	106 (74.1)
ST2, January 2012	1 (0.1)	0	6 (4.2)	137 (95.1)
**Fresh weight (g)**				
ST1, October 2011	1.2 ± 0.1 (0.1–2.6)	(0.3–0.4)	0.7 ± 0.3 (0.2–1.9)	0.5 ± 0.1 (0.3–1.7)
ST1, November 2011	1 ± 0.1 (0.4–2.4)	(0.5–1.2)	0.8 ± 0.1 (0.2–4.2)	0.6 ± 0.1 (0.1–1.6)
ST1, December 2011	1.4 ± 0.3 (1.1–2)	-	0.6 ± 0.1 (0.1–4.6)	0.6 ± 0 (0.1–1.2)
ST1, January 2012	0.7 ± 0.1 (0.3–1.9)	-	0.3 ± 0.0 (0.1–0.7)	0.5 ± 0 (0.1–1.3)
ST2, January 2012	1.2	-	1.8 ± 0.2 (1.3–2.3)	0.5 ± 0 (0.1–1.5)
**Total length (mm)**				
ST1, October 2011	47 ± 3 (19–64)	(26–30)	35 ± 5 (21–53)	44 ± 1 (30–56)
ST1, November 2011	48 ± 2 (38–65)	(31–40)	37 ± 2 (12–70)	36 ± 2 (10–58)
ST1, December 2011	56 ± 4 (51–63)	-	34 ± 2 (17–70)	44 ± 1 (20–56)
ST1, January 2012	40 ± 2 (29–60)	-	30 ± 3 (17–62)	36 ± 1 (4–60)
ST2, January 2012	56	-	58 ± 2 (50–61)	40 ± 1 (27–56)

## Supplementary Materials

The following are available online at http://www.mdpi.com/2072-6651/10/7/275/s1, Figure S1: Sampling sites in Nyanza Gulf, L. Victoria showing cyanobacteria mass accumulation, Figure S2: Relationship between total phytoplankton biovolume (mm^3^ L^−1^) and Chlorophyll *a* (µg L^−1^) for all water samples from different sample types in Kisumu Bay, Nyanza Gulf, and Rusinga Channel, Lake Victoria, Figure S3: Relationship between *Microcystis* cell numbers and total (intracellular and dissolved) MC concentration for all water samples from different sample types in Kisumu Bay, Nyanza Gulf, and Rusinga channel, Lake Victoria, Figure S4: Drying of fish samples for the experiment on microcystin stability (0–8 h) and for the market, Table S1: Phytoplankton taxa which were discriminated for biovolume estimation, Table S2: Meteorological characteristics for dates of fish drying to study the stability of microcystin in fish samples.
