# Supplementary material for: Microcystin Content in Phytoplankton and in Small Fish from Eutrophic Nyanza Gulf, Lake Victoria, Kenya
Source: Toxins (Basel). 2018 Jul 3;10(7):275. doi: 10.3390/toxins10070275 (PMC6070987; doi:10.3390/toxins10070275)
Supplement: Supplementary file 1 [file toxins-10-00275-s001.pdf]

# Supplementary Materials: Microcystin Content in Phytoplankton and in Small Fish from Eutrophic Nyanza Gulf, Lake Victoria, Kenya

**Benard Mucholwa Simiyu, Steve Omondi Oduor, Thomas Rohrlack, Lewis Sitoki and Rainer Kurmayer**

**Table S1.** Phytoplankton taxa which were discriminated for biovolume estimation.

| Genus | Author (Genus) | depth-integrated | surface | patch | shore | Rusinga channel |
|---|---|---|---|---|---|---|
| Cyanobacteria | | | | | | |
| *Anabaena* (*Dolichospermum*) | Bory ex Bornet & Flahault | x | x | x | x | x |
| *Aphanocapsa* | C.Nägeli | | | | | x |
| *Chroococcus* | Nägeli | | | | | x |
| *Cyanodictyon* | A.Pascher | | | | | x |
| *Planktolyngbya* | Anagnostidis & Komárek | x | x | x | x | x |
| *Microcystis* | Lemmermann | x | x | x | x | x |
| *Romeria* | M.Koczwara | | | | | x |
| *Merismopedia* | F.J.F.Meyen | | | | | x |
| Chlorophyceae | | | | | | |
| *Chlamydomonas* | Ehrenberg | x | | | x | x |
| *Coelastrum* | Nägeli | | | | | x |
| *Pediastrum* | Meyen | | | | | x |
| *Scenedesmus* | Meyen | | | | | x |
| *Staurastrum* | Chodat | | | | | x |
| Bacillariophyceae | | | | | | |
| *Aulacoseira* | Thwaites | x | | | | |
| *Nitzschia* | Hassall | | | | | x |
| Cryptophyceae | | | | | | |
| *Cryptomonas* | Ehrenberg | x | x | x | x | x |

X, detected.

**Table S2.** Meteorological characteristics for dates of fish drying to study the stability of microcystin in fish samples (MET Department, Kisumu Airport).

| Date | Air temperature (°C) | Irradiance (μmol photons $m^{-2}$ $s^{-1}$) | Evaporation (mm/day) | Wind speed (knots) (Cumulative: 11.00 am to 5.00 pm) | Relative Humidity (%)[1] |
|---|---|---|---|---|---|
| 27 Oct 2011 | 22.7 | 866 | 4 | 197,084 | 80 |
| 8 Nov 2011 | 24.95 | 1185 | 6.5 | 83,042 | 35 |
| 5 Dec 2011 | 23.75 | 1297 | 3.5 | 145,086 | 78 |
| 9 Jan 2012 | 23.65 | 1238 | 6 | 145,076 | 62 |
| 10 Jan 2012 | 23.6 | 1387 | 6 | 162,056 | 66 |

[1] calculated from temperature, wind speed, and evaporation rate.

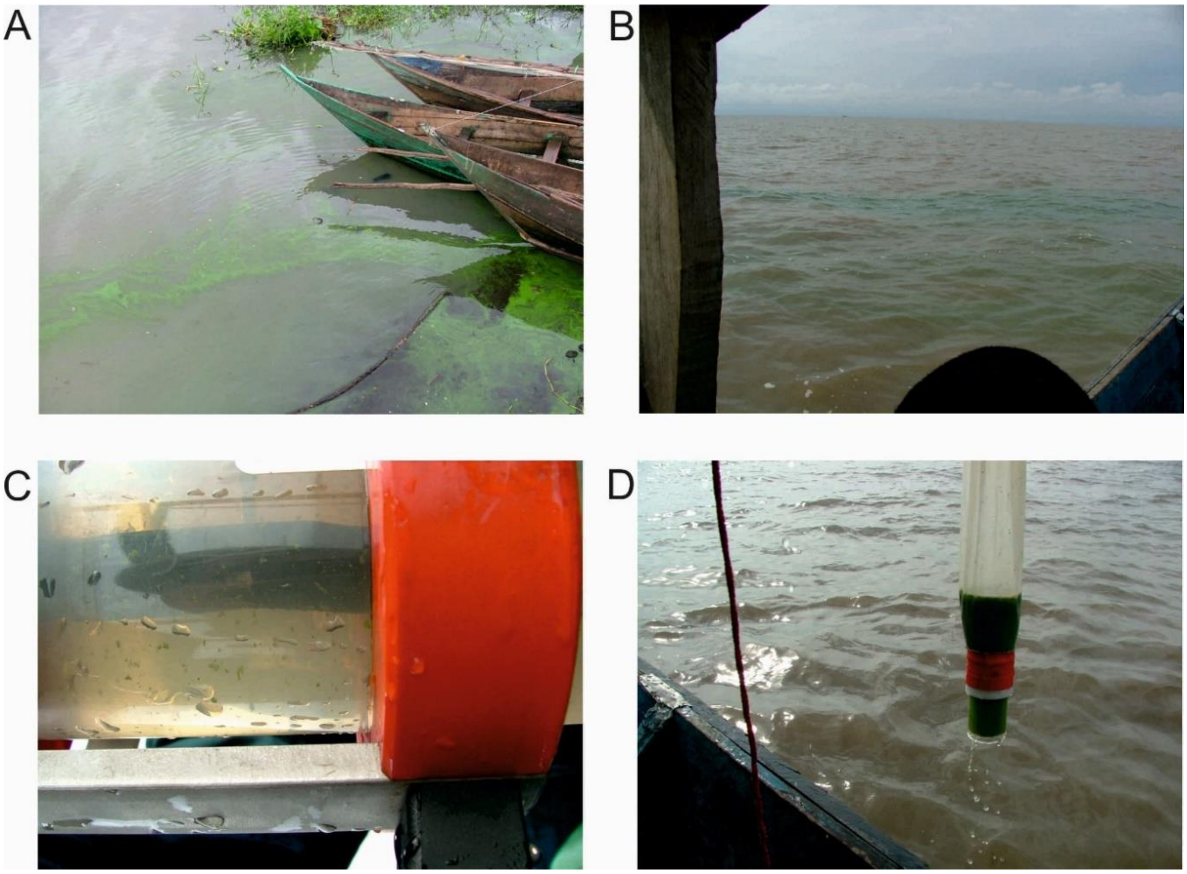


**Figure S1.** Sampling sites in Nyanza Gulf, L. Victoria showing cyanobacteria mass accumulation, **(A)** at the shore of the Dunga beach landing site near Kisumu, **(B)** patches formed by wind action in Kisumu Bay, **(C)** macroscopic colonies of *Microcystis* in the water sample at ST1, **(D)** phytoplankton net sample taken at ST1. The maximum MC concentrations were recorded from shore and patch samples (see Figure 2).

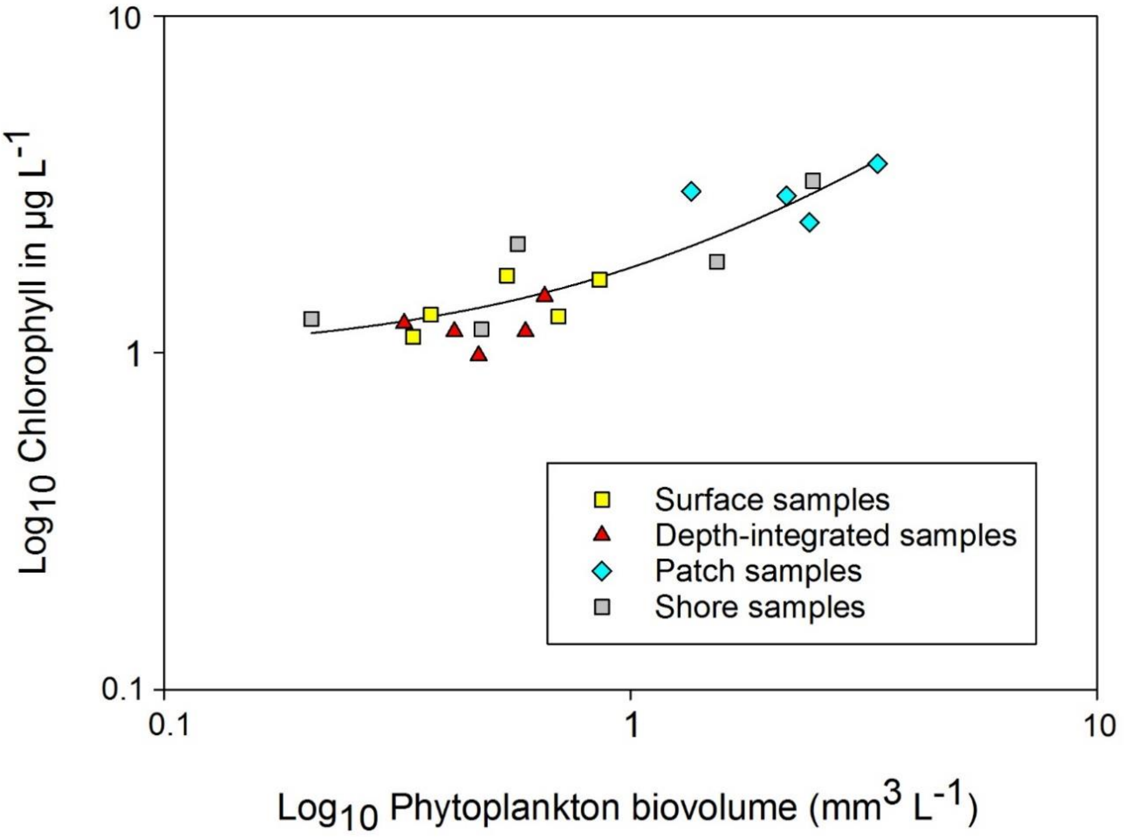


**Figure S2.** Relationship between total phytoplankton biovolume (mm$^3$ L$^{-1}$) and Chlorophyll *a* (µg L$^{-1}$) for all water samples from different sample types in Kisumu Bay, Nyanza Gulf, and Rusinga Channel, Lake Victoria: y = 0.8132x + 0.9757, where y is log$_{10}$ of Chlorophyll a (in µg L$^{-1}$) and x is log$_{10}$ of phytoplankton biovolume (in mm$^3$ L$^{-1}$), ($R^2$ = 0.81), (*n* = 19).

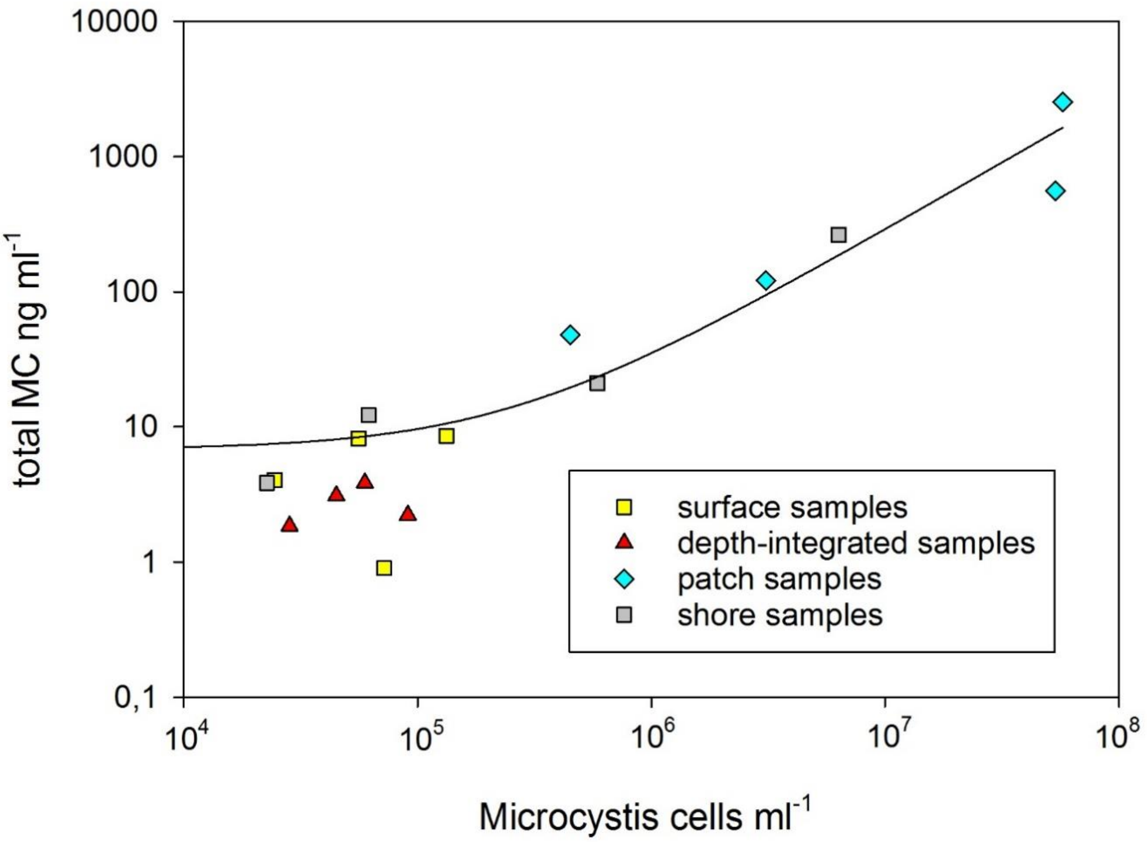


**Figure S3.** Relationship between *Microcystis* cell numbers and total (intracellular and dissolved) MC concentration for all water samples from different sample types in Kisumu Bay, Nyanza Gulf, and Rusinga channel, Lake Victoria: $y = 2.84 \times 10^{-5}x + 6.8044$, where y is MC in ng/ml and x is *Microcystis* cell number/ml ($R^2 = 0.71$), ($n = 19$). No MC was detected in samples from Rusinga channel.

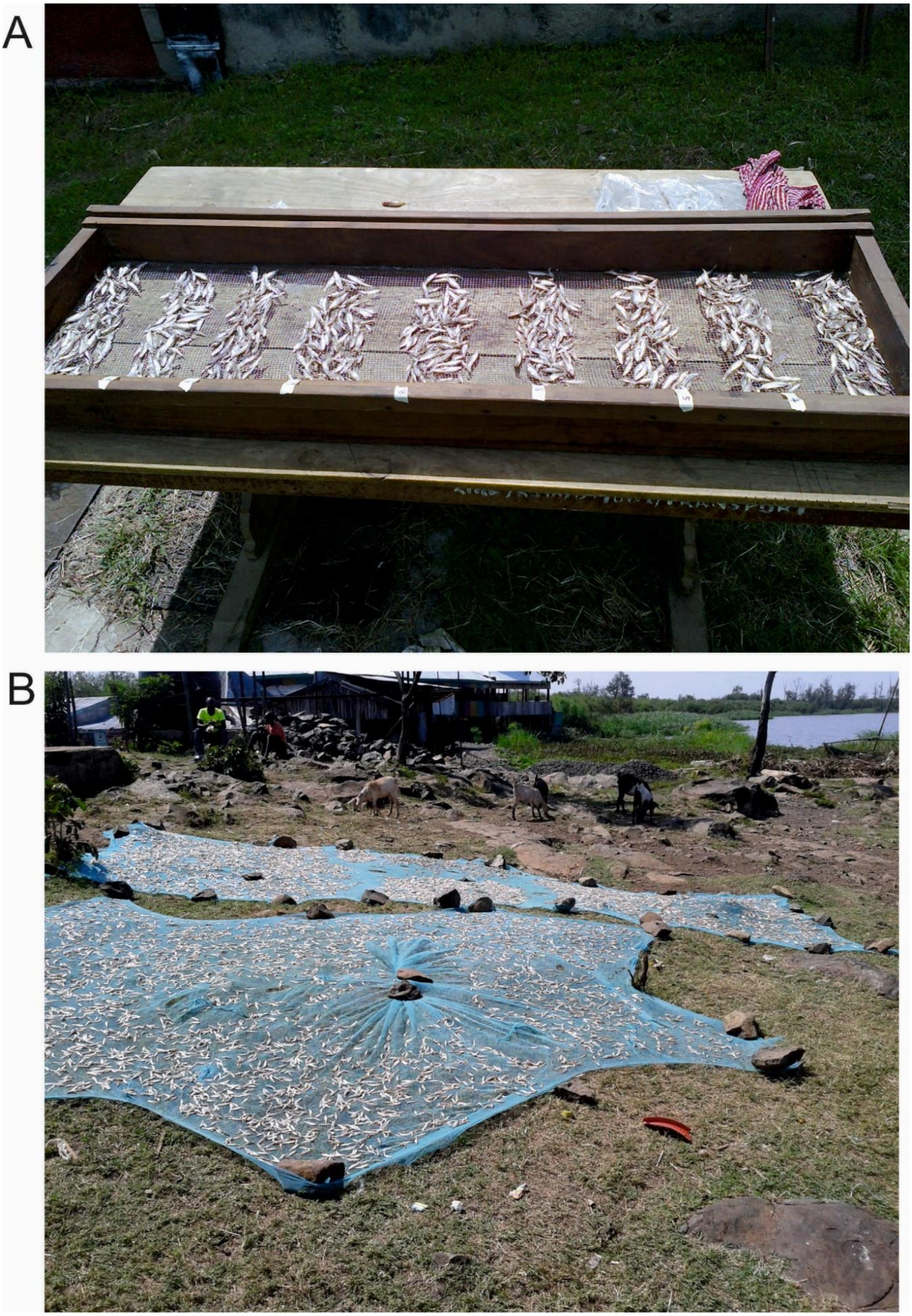

**Figure S4. (A)** Drying of fish samples for the experiment on microcystin stability (0-8 h), **(B)** Drying of fish for the market at the Dunga Beach landing site (Kisumu).
